# Access without excess: a comparison of wound infections with disposable versus hybrid trocars in minimally invasive inguinal hernia repairs

**DOI:** 10.1007/s00464-026-13408-x

**Published:** 2026-10-06

**Authors:** Kimberly P. Woo, Ali Alipouriani, Abby Gross, William C. Bennett, Jenny H. Chang, Alvaro Carvalho, Daphne Remulla, Kayleigh Risser, Nikhila Yerabandi, Li-Ching Huang, Alberto Arezzo, Vittoria Bellato, Myrthe M. Eussen, Maggie Hodges, Sheetal Nijhawan, Marina Yiasemidou, Kimberly S. Miles, Patricia Sylla, Shaneeta M. Johnson, Lucas R. Beffa, Clayton C. Petro, Ajita S. Prabhu, David M. Krpata, Michael J. Rosen, Benjamin T. Miller, SAGES, EAES Sustainability in Surgery Committee

**Affiliations:** 1 Department of Surgery, Center for Abdominal Core Health, Cleveland Clinic Foundation, Cleveland, OH USA; 2 Case Western Reserve University School of Medicine, Cleveland, OH USA; 3 Center for Quantitative Sciences, Vanderbilt University Medical Center, Nashville, TN USA; 4 Division of Gastrointestinal Surgery, Department of Surgery, Northwestern Medicine, Chicago, IL USA; 5 Department of Surgical Sciences, University of Torino, Toriano, Italy; 6 Department of Gastroenterology, Radboud University Medical Centre, Nijmegen, Netherlands; 7 Department of Surgery, Maastricht University Medical Centre, Maastricht, The Netherlands; 8 Department of Surgery, School of Medicine, University of North Carolina at Chapel Hill, Chapel Hill, NC USA; 9 Department of Surgery, Sharon Regional Medical Center, Sharon, PA USA; 10 Colorectal Surgery, The Royal London Hospital, Barts Health NHS Trust, London, UK; 11 Division of Colon and Rectal Surgery, Mount Sinai Hospital, New York, NY USA; 12 Department of Surgery, Meharry Medical College, Nashville, TN USA; 13 Meharry School of Global Health, Meharry Medical College, Nashville, TN USA; 14 Cleveland Clinic Lerner College of Medicine, Cleveland, OH USA

**Keywords:** Surgical sustainability, Hybrid trocars, Reusable trocars, Minimally invasive surgery, Inguinal hernia repair

## Abstract

**Introduction:**

Operating rooms generate 70% of hospital waste. Transitioning from disposable to reusable devices could mitigate surgical waste. Partially reusable, or hybrid, trocars can be used for minimally invasive surgery (MIS), but perceptions of increased infection risk may drive preferences toward disposable trocars. However, no data substantiates this risk. Our analysis of a national hernia outcomes database investigates this presumed association.

**Methods:**

Using the abdominal core health quality collaborative (ACHQC), we identified high-volume inguinal hernia surgeons who used only disposable trocars for laparoscopic inguinal hernia repair (IHR) and hybrid trocars for robotic IHR. These two surgical approaches served as surrogates to evaluate differences between disposable and hybrid trocars. We performed a query of the ACHQC to identify patients who underwent elective MIS IHR with mesh between 2017 and 2023 by one of the previously identified surgeons.

**Results:**

A total of 34,638 patients met inclusion criteria. After propensity score matching, there were 1191 patients in each group. There was no difference in rates of surgical site infection (SSI), surgical site occurrence requiring procedural intervention (SSOPI), readmission or reoperation between the two groups. There was a statistically significant higher rate of surgical site occurrence (SSO) in the disposable trocar group (5% vs. 2%, *p* = 0.003). This was driven by a significantly higher rate of hematomas (12% vs 38%, *p* = 0.007).

**Conclusion:**

This study did not find an increased risk of SSI associated with hybrid trocars compared to disposable trocars for MIS inguinal hernia repairs. To decrease surgical waste, surgeons should consider the use of hybrid trocars for laparoscopic surgery.

Surgical care is a significant contributor of operating room waste, much of which arises from single-use devices and consumables. Efforts to reduce waste increasingly focus on identifying opportunities where reusable or hybrid instruments may be safely substituted for disposable products [1, 2].Technological advances, such as minimally invasive surgery (MIS), have revolutionized surgery with less wound morbidity and faster recovery [3–5]. However, the associated use of additional equipment, devices and consumables have also further increased the carbon footprint of the operating room (OR) [6].

Trocars, required for minimally invasive abdominal access, are an example of one such item. Commonly available trocars can be single-use/disposable, reusable, or hybrid. Hybrid trocars combine a reusable metal cannula with a single-use disposable plastic seal or cap, reducing overall waste while maintaining sterility of components that directly contact tissue. Disposable products are often preferred by hospital systems because there is no need for sterilization or maintenance, and there is a perceived reduction in hospital-acquired infection risk. However, multiple analyses have shown that disposable devices generate substantially more waste and have higher overall cumulative costs than reusable or hybrid systems [6–8]. Disposable trocars must be repurchased for every procedure, whereas hybrid systems distribute cost over multiple uses through repeated sterilization [9–12].

Despite the economic and environmental advantages of reusable trocars, widespread adoption remains limited, possibly due to persistent concerns about infection risks. However, there is no clinical data directly comparing infectious postoperative outcomes between disposable and reusable trocar [13]. To address this knowledge gap, this study aimed to determine whether hybrid trocars have higher rates of surgical site infection compared to disposable trocars in a cohort of patients undergoing MIS inguinal hernia repair.

## Methods

### Data source and patient population

The abdominal core health quality collaborative (ACHQC) registry is a nationwide, prospectively maintained quality collaborative that collects detailed clinical information related to abdominal core health and hernia repairs. Data are entered by participating surgeons or trained abstractors at standardized time points, beginning preoperatively and continuing through long-term follow-up. Variables collected include patient demographics, pre-operative risk factors, intraoperative technical details, postoperative outcomes, and patient-reported outcomes. At the time of this analysis, the registry included data from 458 surgeons and 125,029 patients [12].

Institutional review board approval was obtained through the Cleveland clinic (IRB# 23-1193). The ACHQC registry was then queried for patients undergoing elective, minimally invasive (robotic or laparoscopic) inguinal hernia repair (IHR) with permanent synthetic mesh between January 2017 and December 2023. Cases classified as clean by the centers for disease control and prevention were included. Patients with concomitant ventral hernia repair or other procedures were excluded. Only patients with complete 30-day follow-up were included. The strengthening the reporting of observational studies in epidemiology (STROBE) reporting guidelines were followed [14].

### Exposure variables

The exposure variables were trocar type: disposable versus hybrid. The ACHQC collects data on minimally invasive surgical (MIS) repair approach (e.g., laparoscopic vs robotic) but does not collect data on which trocar type was used. Therefore, we used surrogate variables to conduct our analysis. At our institution, our practice is to use disposable trocars only for laparoscopic IHR (LIHR), and hybrid trocars only for robotic IHR (RIHR). These hybrid trocars consist of a reusable metal cannula and a disposable plastic cap. In this analysis, all LIHRs were assumed to have used disposable trocars and all RIHRs were assumed to have used hybrid trocars, serving as surrogates for their respective trocar types.

Only data from surgeons who follow the previously outlined practice pattern was included in the analysis. To increase the sample size beyond patients at our institution, a survey was distributed to the 15 surgeons within the ACHQC who performed the greatest number of IHRs. The survey asked one question, “Do you use only disposable trocars for your laparoscopic inguinal hernia repairs and only reusable trocars for robotic inguinal hernia repairs?” The analysis was performed using two groups of data from the ACHQC (1) the patients of the surgeons who answered “Yes” to the survey question and (2) the surgeons at the Cleveland clinic who all follow the same practice pattern.

### Outcome of interest

Data on outcomes were collected prospectively in the ACHQC registry and were defined using previously proposed standardized definitions. The primary outcome of interest was surgical site infection (SSI) at 30-day follow-up, including superficial, deep and organ space infections, as defined by the center for disease control and prevention [15]. Secondary outcomes included rate of surgical site occurrence (SSO), SSO requiring procedural intervention (SSOPI), reoperation, and readmission at 30-day follow-up. SSO included SSI and other conditions such as non-healing incisional wounds, skin or soft tissue ischemia, seroma, hematoma, or the development of an enterocutaneous fistula [16]. Procedures classified as SSOPI included wound opening and/or debridement, drainage, or partial or complete mesh removal.

### Propensity score matching

Patients undergoing laparoscopic repair were matched to those undergoing robotic repair using nearest neighbor 1 to 1 propensity score matching (PSM). A caliper width equal to 0.2 of the standard deviations of the logit of propensity scores was used. The variables included in the PS model were as follows: gender (male, female), age (capped at 90, continuous), BMI (capped at 15–60, continuous), current smoker (yes, no), diabetes mellitus (yes, no), chronic obstructive pulmonary disease (yes, no), immunosuppression (yes, no), American Society of Anesthesiologists (ASA) class (1/2, 3/4/5), recurrent hernia as indication for repair (yes, no), and year of operation (2017 to 2023). These variables were selected based on clinical consideration. The standardized mean differences (SMDs) were used to evaluate matching performance, where SMD less than 0.2 is considered acceptable. Due to low missing rates (< 1%), only complete cases were included in PS matching analysis.

### Statistical analysis

Continuous variables were compared using Wilcoxon rank sum test and categorical variables were compared using Pearson Chi-square. A two-sided p-value less than or equal to 0.05 was considered statistically significant. All statistical analyses were conducted using R software (version 4.4, Vienna, Austria).

## Results

### Baseline characteristics

**Fig. 1:**
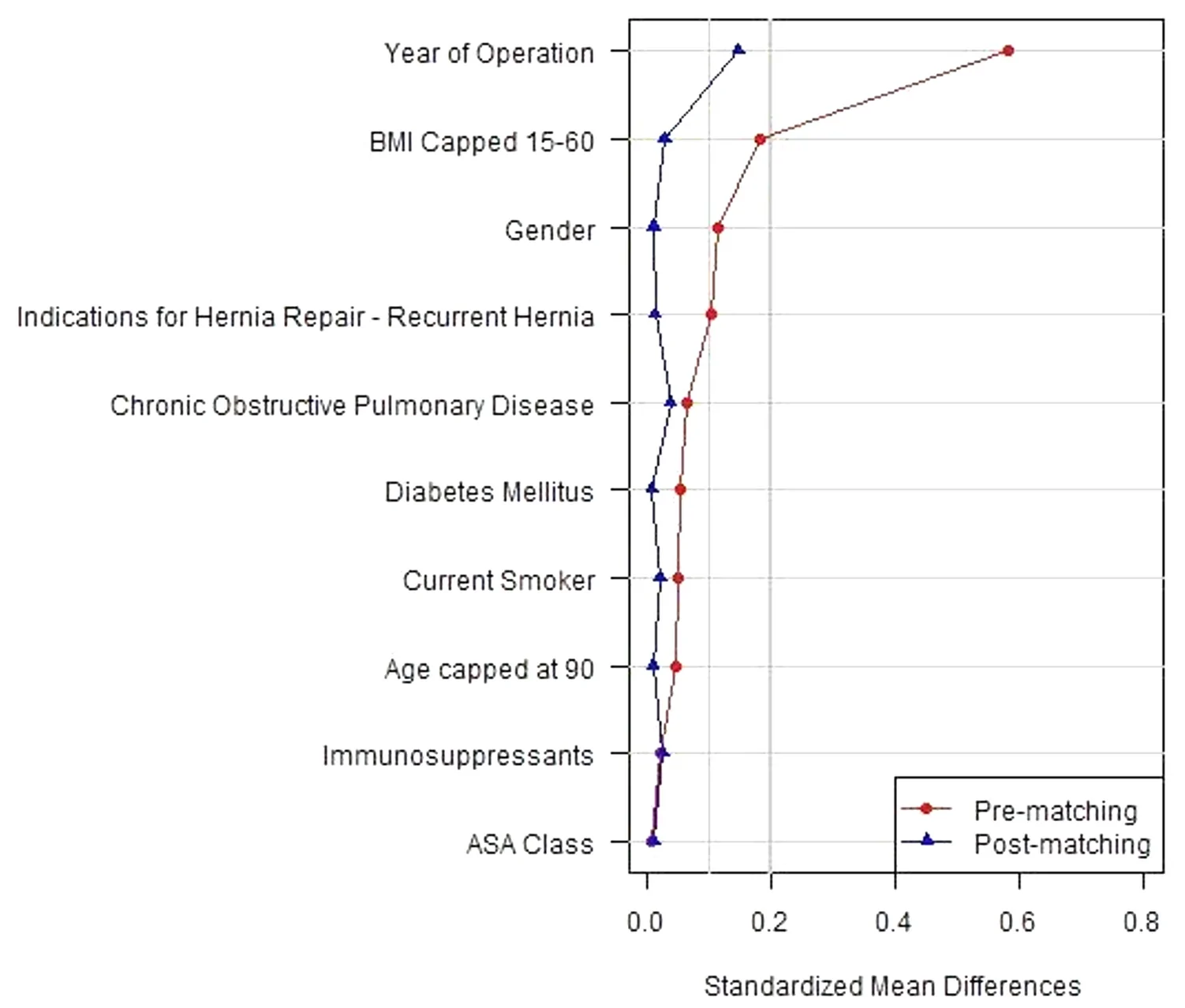
Plot of standardized mean differences before and after propensity score matching between the disposable and hybrid trocar groups

At the time of the query, there were 34,638 patients who underwent IHR in the ACHQC. Of the 15 surgeons to whom the survey was distributed, 13 responded and 11 of those surgeons answered “yes”, indicating that they follow the practice pattern. Data from 17 total surgeons (six at our institution) were included in the analysis. A total of 3166 patients underwent MIS IHR between January 2017 and November 2023 and met the remaining inclusion criteria. Before matching, there was a statistically significant difference between the two groups in gender, BMI, hypertension, year of operation, ASA class, operative time, and recurrent hernia as indication for operative repair. After 1:1 PSM, there were 1191 patients in each group with significant improvement in the balance between the two groups with regard to baseline and operative characteristics (Table 1). All the standardized mean differences were less than 0.2 (Fig. 1).

**Table 1:** Patient baseline and operative characteristics before and after matching

	Unmatched cohort			1:1 Propensity score matched cohort		
	Disposable	Reusable	*P*-value	Disposable	Reusable	*P*-value
	*N* = 1424	*N* = 1742		*N* = 1191	*N* = 1191	
Age capped at 90	64 [53,72]	64 [50,72]	0.5	54 [50,72	63 [51,72]	0.6
Gender			0.0022			0.82
Male	93% (1323)	90% (1563)		92% (1097)	92% (2197)	
Female	7% (101)	10% (179)		8% (94)	8% (185)	
Race			0.7			0.4
White	88% (1258)	89% (1547)		88% (1051)	88% (2088)	
Non-white	12% (166)	11% (195)		12% (140)	12% (294)	
BMI Capped 15–60	26 [24, 28]	26 [24, 29]	<0.001	26 [24, 28]	26 [24, 29]	0.6
Hypertension	35% (502)	39% (685)	0.02	39% (462)	37% (878)	0.05
Diabetes mellitus	6% (83)	7% (124)	0.1	6% (75)	6% (152)	0.9
Chronic obstructive pulmonary disease	2% (30)	1% (23)	0.09	2% (20)	2% (46)	0.4
Anti-coagulation medications	3% (47)	3% (44)	0.2	2% (29)	3% (67)	0.3
Immunosuppressants	1% (17)	1% (25)	0.6	1% (11)	1% (25)	0.5
Current smoker	9% (130)	11% (185)	0.2	9% (112)	10% (231)	0.6
Year of operation			<0.001			0.05
2017	16% (222)	6% (98)		12% (140)	8% (98)	
2018	17% (240)	8% (147)		14% (166)	12% (144)	
2019	17% (248)	11% (198)		15% (183)	16% (191)	
2020	14% (204)	14% (245)		16% (193)	18% (211)	
2021	12% (175)	17% (302)		15% (174)	17% (206)	
2022	12% (165)	23% (407)		14% (165)	14% (168)	
2023	12% (170)	20% (345)		14% (170)	15% (173)	
ASA class			<0.001			0.1
1	17% (241)	12% (205)		16% (195)	13% (155)	
2	65% (928)	70% (1221)		65% (770)	68% (806)	
3	17% (248)	18% (309)		18% (219)	19% (224)	
4	0% (7)	0% (7)		1% (7)	1% (6)	
5	0% (0)	0% (0)		0% (0)	0% (0)	
Operative time, minutes			<0.001			<0.001
0—59	79% (1119)	69% (1201)		78% (933)	68% (807)	
60—119	21% (305)	31% (541)		22% (258)	32% (384)	
> 120	0% (0)	0% (0)		0% (0)	0% (0)	
Indications for hernia repair—recurrent hernia	10% (140)	13% (227)	0.005	10% (122)	11% (126)	0.8
Indications for hernia repair—bowel obstruction	0% (6)	0% (8)	0.9	1% (6)	1% (6)	1

### 30-day outcomes

In propensity score analysis, two patients in the disposable trocar group and one patient from the hybrid trocar group developed SSI (Table 2). There was no difference in rates of SSI between the two groups. There was a higher rate of SSO in the disposable group compared to the hybrid trocar group (5% vs. 2%, *p* = 0.003). Within SSO, the disposable group had a significantly higher rate of seroma (82% vs. 62%, *p* = 0.04) and a lower rate of hematoma (12% vs 38%, *p* = 0.007) (Table 3). There was no difference in rates of SSOPI, readmission or reoperation between the two groups.

**Table 2:** Comparison of 30-day outcomes between disposable versus reusable trocars

	Disposable *N* = 1191	Reusable *N* = 1191	P-value
SSI			0.6
Yes	0% (2)	0% (1)	
No	100% (1189)	100% (1190)	
SSO			0.003
Yes	5% (56)	2% (29)	
No	95% (1135)	98% (1162)	
SSOPI			1
Yes	0% (3)	0% (3)	
No	100% (1188)	100% (1188)	
Reoperation			0.4
Yes	1% (8)	0% (5)	
No	99% (1183)	100% (1186)	
Readmission			0.6
Yes	0% (1)	0% (2)	
No	100% (1190)	100% (1189)

**Table 3:** Surgical site occurrence by complication type

	*N*	Disposable	Reusable	*P*-value
Wound cellulitis	85			0.2
Yes		5% (3)	0% (0)	
No		95% (53)	100% (29)	
Wound serous drainage	85			0.3
Yes		4% (2)	0% (0)	
No		96% (54)	100% (29)	
Wound purulent drainage	85			0.5
Yes		2% (1)	0% (0)	
No		98% (55)	100% (29)	
Seroma	85			0.04
Yes		82% (46)	62% (18)	
No		18% (10)	38% (11)	
Hematoma	85			0.007
Yes		12% (7)	38% (11)	
No		88% (49)	62% (18)	
Unspecified surgical site occurrence	85			0.5
Yes		2% (1)	0% (0)	
No		98% (55)	100% (29)	

## Discussion

In this propensity-matched analysis, we found no evidence that hybrid trocars increase rates of SSI, SSO, or SSOPI compared with disposable trocars, even in patients receiving permanent mesh. Although disposable trocars are commonly used for laparoscopic IHR, the reasons for this preference are unclear. Robotic surgeons, who use hybrid trocars out of necessity, routinely report low infection rates, and our findings similarly show no increase in postoperative morbidity. These results suggest that hybrid trocars can be safely incorporated into laparoscopic practice, though prospective comparative studies are still needed.

Concerns regarding reusable or hybrid trocars typically stem from studies demonstrating high microbial loads on instruments [14, 17–19], or rare infections related to decontamination failures [20–27]. However, modern evaluations show that true sterilization breaches are uncommon [28], validated processes are highly effective [29], and no clinical studies have linked sterilization failure to postoperative SSI. Our findings therefore provide clinical evidence that counters theoretical contamination concerns and align with infection-prevention guidelines, including AORN and WHO [30], which affirm the safety of established sterilization protocols.

Beyond these aforementioned analyses, studies directly comparing clinical infectious outcomes between disposable and reusable surgical materials are limited. Our review of the literature did not identify any studies which specifically compare clinical infections for disposable versus reusable trocars, despite their routine use in minimally invasive surgery. Within their systematic review comparing disposable versus reusable instruments, Eussen et al. found no studies comparing the contamination risk between disposable and reusable instruments [31]. The paucity of data within the literature underscores the value of our results which is the first to compare the clinical infectious outcomes in a large cohort of patients and found no differences in rates of wound infections between the two trocar types.

The environmental impact of disposable versus reusable surgical instruments has been well documented [7, 9, 10, 13, 32, 33]. Life-cycle analyses consistently show that disposable devices generate substantially more waste and resource use than reusable systems. For trocars specifically, Boberg et al. found that reusable systems had the lowest environmental impact across all analyses, while disposable trocars produced several-fold greater emissions. Rizan et al. similarly demonstrated lower environmental impact across human health, ecosystem, and resource categories for hybrid compared with disposable trocars. These findings align with studies of other medical devices, including laryngoscopes, scissors, blood pressure cuffs, gowns, cystoscopes, and anesthetic trays, which have also shown lower environmental impact with reusable alternatives [12, 34–38].

Many environmental analyses have also incorporated cost, consistently showing long-term savings with reusable instruments. Early cost evaluations in the 1990s and 2000s demonstrated meaningful per-case savings when reusable devices were adopted [7, 19, 33, 39]. A more detailed analysis by Ransom et al. specifically examining trocars found that, when accounting for instrument lifespan, maintenance, sharpening, sterilization, and repackaging, disposable trocars resulted in a higher cost per procedure [40]. More recent evaluations—reflecting current pricing, similarly report that reusable trocar systems are approximately half as expensive as disposable systems over their lifetime [9]. Although disposable trocars have lower upfront purchase costs, cumulative costs associated with repeated repurchase exceed those of reusables.

Despite their advantages, reusable devices remain underused because they are often perceived as less convenient to integrate into routine practice. Stakeholder interviews by Davies et al. highlight that additional steps in cleaning, processing, and procurement, along with competing institutional priorities, contribute to hesitant adoption [41–43]. Vendor contracts and supply-chain constraints can further limit flexibility. Finally, perception barriers surrounding the sterilization and safety of reusable instruments have been described in the literature as additional contributors to limited uptake in some settings. Successful transition to reusable systems will require institutional support and processes that make their use as seamless as disposable options.

## Limitations

This study has several important limitations. Trocar type was inferred using laparoscopic (disposable) versus robotic (hybrid) approaches, and although literature suggests similar wound morbidity between these techniques, using operative approach as a surrogate introduces potential misclassification and residual confounding. Surgeon inclusion relied on voluntary survey responses, which may introduce response bias. Because the survey did not ask why surgeons do or do not use hybrid trocars, we could not assess factors such as surgeon preference, instrument availability, or usability, representing a missed opportunity and a major unmeasured factor. Although propensity score matching reduced baseline differences, unmeasured confounders may still influence outcomes. In addition, reusable hybrid trocars are often metallic, and any potential differences in risks related to surgical energy use were not evaluated in the present study. Generalizability is limited because ACHQC surgeons are hernia specialists, and findings may not extend to other practice environments. Finally, results apply only to elective, clean inguinal hernia repairs, and may not be applicable to other laparoscopic procedures with different operative characteristics or infection profiles.

## Conclusion

Hybrid trocars were not associated with higher infectious wound morbidity compared with disposable trocars in MIS inguinal hernia repair. Hybrid trocars are already routine in robotic IHR, and our results indicate that they can be safely adopted in laparoscopic IHR without increasing postoperative infection risk. Prospective studies across additional laparoscopic operations may further inform broader adoption and support sustainable instrument selection.

## Data Availability

The data used in this study are not publicly available but can be provided upon reasonable request.
